# Next-Generation Survey Sequencing and the Molecular Organization of Wheat Chromosome 6B^¶^

**DOI:** 10.1093/dnares/dst041

**Published:** 2013-10-01

**Authors:** Tsuyoshi Tanaka, Fuminori Kobayashi, Giri Prasad Joshi, Ritsuko Onuki, Hiroaki Sakai, Hiroyuki Kanamori, Jianzhong Wu, Hana Šimková, Shuhei Nasuda, Takashi R. Endo, Katsuyuki Hayakawa, Jaroslav Doležel, Yasunari Ogihara, Takeshi Itoh, Takashi Matsumoto, Hirokazu Handa

**Affiliations:** 1 Bioinformatics Research Unit, National Institute of Agrobiological Sciences, Tsukuba 305-8602, Japan; 2 Plant Genome Research Unit, National Institute of Agrobiological Sciences, Tsukuba 305-8602, Japan; 3 Laboratory of Plant Genetics, Graduate School of Agriculture, Kyoto University, Kyoto 606-8502, Japan; 4 Centre of the Region Haná for Biotechnological and Agricultural Research, Institute of Experimental Botany, CZ-78371 Olomouc, Czech Republic; 5 Nisshin Flour Milling, Inc., Tsukuba 300-2611, Japan; 6 Kihara Institute for Biological Research, Yokohama City University, Yokohama 244-0813, Japan

**Keywords:** wheat, chromosome 6B, genome sequencing, next-generation sequencing

## Abstract

Common wheat (*Triticum aestivum* L.) is one of the most important cereals in the world. To improve wheat quality and productivity, the genomic sequence of wheat must be determined. The large genome size (∼17 Gb/1 C) and the hexaploid status of wheat have hampered the genome sequencing of wheat. However, flow sorting of individual chromosomes has allowed us to purify and separately shotgun-sequence a pair of telocentric chromosomes. Here, we describe a result from the survey sequencing of wheat chromosome 6B (914 Mb/1 C) using massively parallel 454 pyrosequencing. From the 4.94 and 5.51 Gb shotgun sequence data from the two chromosome arms of 6BS and 6BL, 235 and 273 Mb sequences were assembled to cover ∼55.6 and 54.9% of the total genomic regions, respectively. Repetitive sequences composed 77 and 86% of the assembled sequences on 6BS and 6BL, respectively. Within the assembled sequences, we predicted a total of 4798 non-repetitive gene loci with the evidence of expression from the wheat transcriptome data. The numbers and chromosomal distribution patterns of the genes for tRNAs and microRNAs in wheat 6B were investigated, and the results suggested a significant involvement of DNA transposon diffusion in the evolution of these non-protein-coding RNA genes. A comparative analysis of the genomic sequences of wheat 6B and monocot plants clearly indicated the evolutionary conservation of gene contents.

## 1. Introduction

Common wheat, also known as bread wheat (*Triticum aestivum* L.), is a major staple food crop in many parts of the world; therefore, there is a strong demand for the genetic improvement of wheat to achieve better quality, higher yield, adaptation to various environments, and tolerance to biotic stresses. These improvements would contribute significantly to human welfare. Highly detailed genomic information is an important tool for the genetic improvement of wheat, but the full sequencing of the wheat genome has been challenging.

Wheat has a large genome, of ∼17 Gb, and is allohexaploid, with three homoeologous genomes (2*n* = 6*x* = 42, genome formula AABBDD) that have been suggested to originate from *Triticum urartu* (2*n* = 2*x* = 14, AA) as a donor of the A genome, *Aegilops tauschii* (2*n* = 2*x* = 14, DD) as a donor of the D genome, and *Aegilops speltoides* (2*n* = 2*x* = 14, SS) or a related species as a possible donor of the B genome, although the identity of the B genome donor is still debated.^1,2^ Additionally, ∼80–90% of the wheat genome is composed of repetitive sequences,^3^ which is a significantly higher percentage than *Brachypodium* (22%), rice (26%), and sorghum (54%).^4,5^ The large size and polyploidy-related complexity of the wheat genome have hampered genomic analysis, and decoding of the whole genome remains challenging, even though next-generation sequencing (NGS) technology has recently been applied.^6^

Chromosome sorting by flow cytometry can reduce sample complexity and simplify the sequencing of complex genomes by dividing these genomes into smaller parts.^7^ Using this method, survey sequences of individual chromosome (1H) or chromosome arms (2HS-7HL) in the barley genome have been obtained and analysed with NGS technology.^8,9^ In wheat, the sorting of single chromosomes or chromosome arms from the cultivar ‘Chinese Spring’ (CS) and its aneuploid lines has enabled the construction of chromosome (arm)-specific BAC libraries,^10^ and these BAC libraries have served as the critical resources for the development of physical maps and map-based genome sequencing by the International Wheat Genome Sequencing Consortium (IWGSC; http://www.wheatgenome.org/, 20 September 2013, date last accessed). As in barley, the sequencing of several wheat chromosomes (1A, 1B, 1D, 3B, 4A, and 5A) or chromosome arms (1AL, 3AS, 7BS, and 7DS) has been conducted using NGS.^11–19^ These survey sequences from whole-chromosome shotgun sequencing are highly informative, bringing not only insights into the molecular organization and evolution of the wheat genome at an unsurpassed resolution but also detailed contents of the syntenic genes among grass genomes.^14–16^ Recently, whole-genome shotgun sequence analysis was performed on the two diploid progenitors of wheat, *T. urartu* and *Ae. tauschii*, which provided information that was useful for decoding the complex polyploid nature of the wheat genome.^20,21^

Chromosome 6B is the third largest chromosome in common wheat, with a total molecular size of 914 Mb, representing 5.4% of the wheat genome. Chromosome 6B consists of a 415 Mb short arm (6BS) and a 498 Mb long arm (6BL).^10^ Similar to chromosome 1B, chromosome 6B can be distinguished from other wheat chromosomes in the karyotype by the presence of a satellite with a secondary constriction on the short arm.^22^ A translocation of chromosomal segments from 6BS and 2BS has also been reported.^23^ Moreover, a pericentromeric inversion has been observed in the cultivar CS.^23,24^ These structural features differentiate the chromosome 6B from the homoeologous chromosomes 6A and 6D.

Up to 30 loci, including genes underlying agronomic, morphological, and physiological traits, have been genetically mapped to the wheat chromosome 6B.^25^ Among these genes, only two gene loci, *Nor-B2* (nucleolus organizer region) and *Gli-B2* (α/β-gliadin seed storage protein), have been studied well. The *Nor-B2* locus, which is located in the secondary constriction of 6BS, contains approximately 5500 copies of the rRNA genes,^26^ and the *Gli-B2* locus is located in a position distal to the *Nor-B2* locus, within ∼10 cM of the 6BS satellite region.^27^ A recent sequencing study of the *Gil-B2* locus mapped to chromosome 6B revealed that 11 α/β-gliadin genes, including one pseudogene, were clustered within an ∼260 kb genomic region.^28^ In tetraploid wheat, two agronomically important genes on chromosome 6B, *Gpc-B1* (grain protein content) and *Yr36* (wheat stripe rust resistance), were isolated using a map-based cloning strategy.^29,30^

In this study, we conducted whole-chromosome shotgun sequencing using DNA amplified from the flow-sorted chromosome arms 6BS and 6BL, which were derived from a double-ditelosomic 6B (dDt6B) line of CS. The DNA samples of 6BS and 6BL were sequenced with a long-read-type NGS (Roche 454 GS-FLX Titanium). The assembled sequences were analysed to characterize the genomic composition of wheat chromosome 6B, including its gene and repetitive sequence contents and its syntenic relationship with other grass genomes, and to identify microRNA (miRNA) and tRNA precursors. These data will be useful for developing new 6B-specific molecular markers to construct BAC-based physical maps and future molecular breeding with marker-assisted selection, which will increase the understanding of the evolutionary and functional aspects of the wheat genome.

## 2. Materials and methods

### 2.1. Plant materials

Seeds of dDt6B of the hexaploid wheat cultivar CS (accession number LPGKU2269) were obtained from the National BioResource Project of Japan (http://www.shigen.nig.ac.jp/wheat/komugi/top/top.jsp, 20 September 2013, date last accessed). The dDt6B line was originally developed by Sears,^31^ and this line contains chromosome 6B as a pair of telosomes, of which one is a short arm (6BS), and the other is a long arm (6BL). The karyotype (20″ + t″6BS + t″6BL) was confirmed by C-banding.^32^

### 2.2. Chromosome sorting and DNA amplification

Liquid suspensions of intact mitotic chromosomes were prepared from synchronized root tips. The samples were stained with 2 μg/ml 4′,6-diamidino-2-phenylindole (DAPI), and the telosomes were sorted using a FACSVantage SE flow cytometer (Becton-Dickinson, San Jose, USA). The level of purity in the sorted fractions was determined by fluorescence *in situ* hybridization (FISH) according to the method described by Kubaláková *et al*.^33^ The DNA of the sorted chromosome arms was purified as described by Šimková *et al*.^34^ and then amplified by multiple displacement amplification using the illustra™ GenomiPhi V2 DNA Amplification Kit (GE Healthcare Bio-Sciences Corp., Piscataway, NJ, USA). The amplified DNA was purified by ethanol precipitation before sequencing.

### 2.3. NGS and assembly

The chromosome arm-specific DNA from 6BS and 6BL was sequenced independently using the 454 GS-FLX Titanium (Roche, CT, USA) at Hokkaido System Science Co., Ltd (Sapporo, Hokkaido, Japan) and Takara Bio, Inc. (Otsu, Shiga, Japan), respectively. The 454 sequenced read data reported here have been deposited in the DNA Data Bank of Japan (DDBJ) Sequence Read Archive (DRA) and are available under accession number (DRA000979).

The sequence reads from each arm were assembled using a GS assembler 2.7 (Roche) with the parameter ‘-large −vt’ to remove the vector sequence. The assembled contigs were compared with the registered sequences of the human genome and the non-redundant database in the DDBJ/EMBL/GenBank by BLASTN with the threshold of *E*-value < 10^−5^. Contigs with human genomic sequence or other non-plant (non-Viridiplantae) sequences as the best hit were removed from subsequent analysis.

### 2.4. Detection of repeats and genes for functional RNA species

Repeat regions were detected with Censor (http://www.girinst.org/censor/index.php, 20 September 2013, date last accessed)^35^ with the option ‘-mode norm’. In addition to an existing repeat library, TREP complete annotation (http://wheat.pw.usda.gov/ITMI/Repeats/, 20 September 2013, date last accessed), a *de novo* repeat family constructed using RepeatModeler (http://www.repeatmasker.org/RepeatModeler.html, 20 September 2013, date last accessed), was used for repeat detection. To detect ribosomal DNA (rDNA) regions, a homology search against unmasked contigs using BLAT was performed with the options ‘-fine -q = rna –out = blast’ and thresholds of ≥95% identity and ≥100 bp coverage. As queries, four rDNA sequences, 5S (3IZ9), 5.8S (3IZ9), 18S (3IZ7), and 25S (3IZ9), and one spacer region between the 25S and 18S rDNAs (X07841) were downloaded from DDBJ/EMBL/GenBank (http://www.ddbj.nig.ac.jp/, 20 September 2013, date last accessed).

The tRNA genes were predicted using the tRNAscan-SE ver. 1.3.1 program.^36^ The tRNA genes in *Brachypodium distachyon*, rice, and sorghum were also predicted using the same procedure. Any tRNAs that were annotated as ‘possible pseudogenes’ were not counted.

We performed the miRNA prediction following the procedure in the previous report for wheat chromosome 5A.^13^ Mature and immature plant miRNAs were downloaded from miRBase (http://www.mirbase.org/, 20 September 2013, date last accessed).^37^ In total, 4677 miRNAs from 193 different organisms were available. First, using mature miRNAs as query sequences, BLASTN was performed against the assembled sequences with the option *E*-value of 10 and a word size of 7. If a mature miRNA showed hits with direct and reverse orientation within a contig, then the existence of an immature miRNA was postulated. Because the BLASTN hits did not always cover an entire query sequence, hit regions were extended in the 5′/3′ direction, and the number of mismatches between a query and a hit region was recalculated using the ClustalW program. Two or fewer mismatches in at least one strand (direct/reverse) were accepted. Then, after an extension of 13 bp at both edges,^38^ immature miRNAs with lengths of ≥1000 bp were discarded because the longest immature miRNA in the known miRNAs of the plants we used is shorter than 1000 bp. Finally, the secondary structure of the immature miRNA was predicted with UNAfold 3.2.^39^ The minimal folding free energy index (MFEI) was calculated for each structure using the following equation: MFEI = AMFE/(G + C)%, where the adjusted MFE (AMFE) is the minimal free energy of 100 nucleotides. All sequences with an MFEI of ≥0.85 were accepted as miRNAs.^40^

### 2.5. Gene annotation

In this study, we determined the expressed loci using two methods: FLcDNA/mRNA mapping and *ab initio* gene prediction with EST evidence. These methods were developed for the rice genome annotation (The Rice Annotation Project).^41^ Wheat FLcDNAs were downloaded from TriFLDB (http://trifldb.psc.riken.jp/index.pl, 20 September 2013, date last accessed),^42^ and the mRNAs and ESTs were retrieved from DDBJ/EMBL/GenBank with the keyword ‘*Triticum aestivum*’. FLcDNAs/mRNAs were processed by removing the poly-A sequences and repeat masking by Censor using the TREP complete annotation with the option ‘-mode norm’. Then, processed FLcDNAs/mRNAs with lengths of >29 bp after removal of the repeated regions were used for further transcript mapping.^41^ These sequences were mapped on assembled contigs using BLAST+ with the parameters ‘-task blastn -evalue 0.01 -lcase_masking’, and with est2genome in the EMBOSS package with the parameters ‘-align -mode both -gappenalty 8 -mismatch 6 -minscore 10’. Transcripts mapped to a contig with >95% identity and >90% cumulative coverage were accepted. Mapped regions that were masked >50% by repeat sequences were discarded from further analysis. To define the transcribed regions (loci), mapped transcripts in exonic regions with at least one base of overlap were clustered.^41^

*Ab initio* gene prediction was performed with the AUGUSTUS program^43^ trained using the rice build 5 annotation data.^44^ If the predicted genic regions were relatively less masked regions (<50%), then the predicted genes were classified as ‘non-repetitive genes’. As expression evidence, we used ESTs mapped by BLASTN with ≥95% identity and ≥90% coverage on a contig.^41^ Regions that overlapped by at least one base with EST-mapping regions were defined as ‘proven predicted genes’.

### 2.6. Detection of orthologues in cereals

Rice annotation data were retrieved from RAP-DB (http://rapdb.dna.affrc.go.jp/, 20 September 2013, date last accessed),^44^ and the data for *B. distachyon* and sorghum were downloaded from Phytozome (http://www.phytozome.net/, 20 September 2013, date last accessed).^4,45^ Barley high-confidence genes were downloaded from MIPS PlantsDB (http://mips.helmholtz-muenchen.de/plant/barley/index.jsp, 20 September 2013, date last accessed).^46,47^ First, all genes from the four species were mapped on the combined contigs of 6BS and 6BL by tBLASTN using the parameter ‘-e 10-5 –U T’. Second, the best pairs between a gene and a contig were selected with the top hit of the BLAST search. Third, mapped genes with 1 bp overlap were clustered on contigs.^41^

## 3. Results and discussion

### 3.1. Chromosome arm sorting and DNA preparation

**Figure 1.**
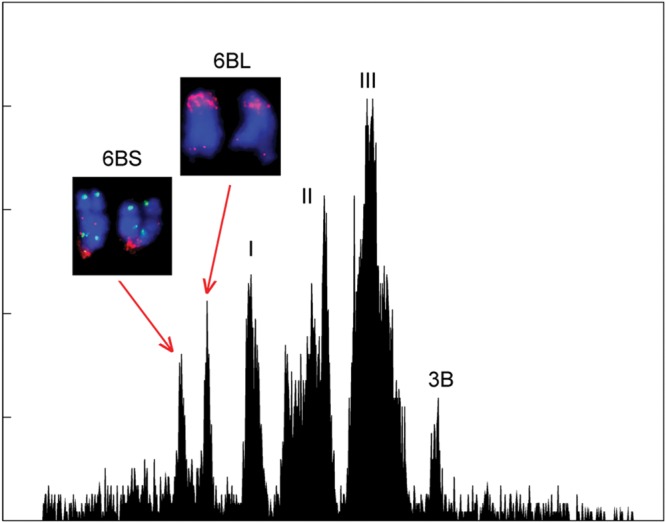
The histogram of the relative fluorescence intensity (flow karyotype) obtained from the flow cytometric analysis of DAPI-stained mitotic metaphase chromosomes isolated from a double-ditelosomic line 6B of the common wheat cultivar CS. The histogram consists of a chromosome 3B peak, a small composite peak I containing chromosomes 1D, 4D, and 6D, and two large composite peaks, II and III, containing the remaining 16 chromosomes. The two additional peaks represent the short-arm telosome 6BS and the long-arm telosome 6BL, which can be easily discriminated and sorted. The two telosomes can be identified by FISH with GAA microsatellite (red) and Afa repeat (green) probes (insets). *X*-axis: relative DAPI fluorescence intensity; *Y*-axis: number of particles.

Both of the chromosome arms of wheat chromosome 6B were sorted as telocentric chromosomes 6BS and 6BL from a double-ditelosomic line (dDt6B) by flow cytometry (Fig. 1). The use of telosomic stocks and flow cytometric sorting permits the dissection of the large wheat genome into small and well-defined pieces, facilitating analysis and mapping.^7^ Chromosome arms 6BS and 6BL were flow-sorted in batches of 59,000 and 49,000, respectively, from approximately 40,000 seeds obtained from 50 dDt6B plants, and the average purity in the sorted fractions, as estimated by FISH, was 91.2 and 92.8% for 6BS and 6BL, respectively. The approximately 7–9% contamination was due to a mixture of other chromosomes. Chromosomal DNA was extracted and amplified in six independent multiple displacement amplification reactions. Finally, we obtained ∼20 µg of amplified DNA from each chromosome arm for use in 454 shotgun sequencing.

### 3.2. Shotgun sequencing and assembly of chromosome 6B arms

The sequencing details representing the main metrics of the 454 sequencing and the assemblies for 6BS and 6BL are summarized in Table 1. Because the estimated lengths of 6BS and 6BL were 415 and 498 Mb, respectively,^10^ our total read lengths, 4.94 Gb for 6BS and 5.51 Gb for 6BL, were equivalent to a sequencing depth of ∼11.9- and 11.1-fold, respectively. After the sequence assembly and the removal of short contigs (<200 bp), the total lengths of the assembled contigs for 6BS and 6BL were 234.8 and 273.2 Mb, comprising 262 375 and 173 655 contigs, respectively, which corresponds to 56.6 and 54.9% of the estimated lengths of both arms. These total lengths of the assembled contigs and the coverages of the estimated chromosome size were larger than those reported for other chromosome arms: 60.9 Mb (20.6%) and 116.2 Mb (21.8%) for 5AS and 5AL, respectively^13^; and 146.7 Mb (46.3%) and 239.6 Mb (44.5%) for 4AS and 4AL, respectively.^16^ Although the number of short contigs (<200 bp) is relatively large, 43 618 and 21 978 for 6BS and 6BL, these contigs are equivalent to only 6.41 and 3.23 Mb for 6BS and 6BL, respectively, which indicates that the effect of these contigs on the total assembled length is limited.

**Table 1.** The summary of 454 sequencing reactions and assemblies for the short and long arms of chromosome 6B

	6BS	6BL
Total reads	12 873 283	12 082 150
Total bases (bp)	4 941 174 940	5 507 636 827
Average read length (bp)	383.83	456.00
Average quality value	27.6	27.3
Number of contigs	262 375	173 655
Total bases (bp)	234 772 755	273 193 549
N50 (bp)	1107	2675
Min (bp)	200	200
Max (bp)	24 902	38 754
Mean (bp)	894.8	1573.2
Average depth (reads/contig)	9.05	10.1
Median depth (reads/contig)	5.2	6.7

### 3.3. Repeated structure of wheat chromosome 6B

The wheat genome is composed of abundant repetitive elements, and >80% of the genome is occupied by repeated sequences.^3,13,15,48^ Using the known TREP library and the *de novo* repeat library from this study, we determined that 76.6% of the 6BS assembly and 85.5% of the 6BL assembly correspond to repeat elements. More than 13% of the repetitive regions in the assemblies of both chromosome arms were masked only in the *de novo* repeat library. These results indicate that chromosome 6B contains novel, unannotated repeat sequences, providing important insight into the genomic structure of wheat chromosomes for future reference genomic sequencing by the IWGSC.

**Figure 2.**
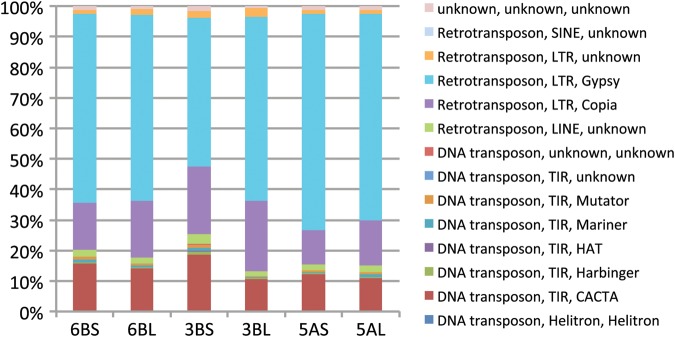
The distribution of repetitive elements in wheat chromosomes 3B, 5A, and 6B. Repeat detection in chromosomes 3B^11^ and 5A^13^ was conducted by the same procedure used for chromosome 6B. Only the TREP repeat data were used to categorize repetitive elements.

We used the TREP library to further classify the contigs that matched known transposable elements (TEs) into TE families (or subfamilies) according to the categories of a unified classification.^49^ We excluded the repeated contigs in the *de novo* library from this classification because these contigs were not well annotated and therefore could not be integrated under the same criteria as the contigs in the TREP library. We did not observe any bias in the distribution of the repetitive elements along each arm of 6B (Fig. 2). The LTR/Gypsy family was most frequent in chromosome 6B, followed by the LTR/Copia and DNA transposon CACTA families. This distribution tendency observed in 6B is essentially conserved in other sequenced wheat chromosomes (Fig. 2).

**Figure 3.**
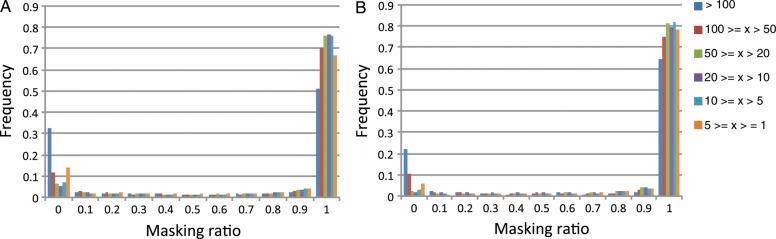
The relationship between the masking ratio and the read depth of the contigs located in the short arm (A) and long arm (B) of chromosome 6B.

**Figure 4.**
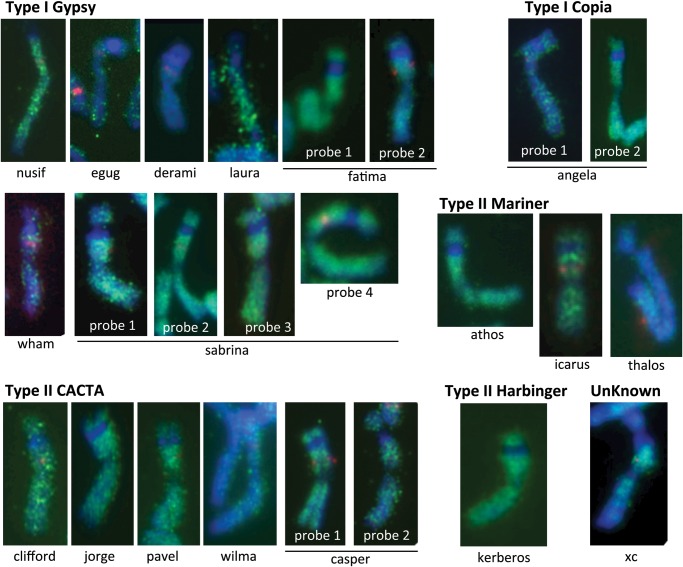
The distribution patterns of the TEs on chromosome 6B. Chromosome 6B was identified by the red AG_12_ signal, and the distributions of the transposons are represented by the green signals. The chromosomes are arranged with the short arms on top. The transposon probes displayed uniform labelling of chromosome 6B, with the exception of the satellite and centromeric regions.

Previous survey sequencing of wheat chromosome 5A revealed that most of the contigs with high coverage rates consisted of repeated sequences.^13^ However, highly masked contigs do not always have higher read depths, as shown in Fig. 3, and contigs with lower read depths were sometimes repetitive. We assume that repeat sequences are not always the cause of genome degeneration in the genomic assembly, and more precise analysis is necessary for an accurate conclusion. We used FISH to obtain insight into the distribution of TEs along chromosome 6B. We selected 24 families of TEs representing major components of the transposons found in our assemblies (Fig. 2). We amplified the unique regions of these TEs using PCR (Supplementary Table S1); 20 samples yielded products of the expected size. In two cases (Thalos and Icarus), the fragment size was smaller than expected, and the primers for Jorge and Athos did not produce discrete bands by agarose gel electrophoresis; there were smear patterns for Jorge and multiple bands for Athos. FISH with a probe for the AG_12_ microsatellite was used to identify chromosome 6B, which has the third strongest signal in the interstitial region of the short arm (Supplementary Fig. S1). Chromosome 6B can be discriminated from chromosome 3B by the presence of satellite DNA on the short arm. The distribution patterns of transposons were represented by FISH signals with each probe, as shown in Fig. 4. All of the tested probes displayed dispersed localization patterns along the length of chromosome 6B. Notably, the NOR (nucleolus organizer region) and centromere regions lacked transposon FISH signals. We did not observe striking differences in the distribution patterns of the transposons, and these patterns were not dependent on transposon family, class, or type. Notably, the TE sequences were not restricted to the C-band/N-band-positive pericentromeric heterochromatin. We found that the distribution of some transposons were not ubiquitous in the hexaploid wheat complement (Supplementary Table S1).

### 3.4. Detection of transcribed regions

More than 80,000 FLcDNAs/mRNAs and millions of ESTs are available in the DDBJ/EMBL/GenBank and TriFLDB public databases, facilitating wheat transcriptome analysis.^42^ To detect the transcribed regions and to annotate the gene structures on chromosome 6B, we mapped these transcribed sequences to our assembled contigs. Using the two methods (the transcript mapping and *ab initio* gene prediction) described extensively in Materials and methods, we identified 2032 and 2766 loci on 6BS and 6BL, respectively, as genic regions supported by the data from the wheat transcriptome (Table 2), comparable with the results for chromosomes 3B and 5A.^11,13^

**Table 2.** The statistics of the transcriptomes in the short and long arms of chromosome 6B

	Number of transcripts	Number of trimmed transcripts	6BS	6BL
FLcDNA/mRNAs	84 164	83 425	2703 (2238)*	4754 (3494)*
50% ≥ masking			1762 (1343)*	3016 (1851)*
EST	1 286 173	1 281 733	48 695	53 315
Predicted genes			4967	5613
Overlapping with ESTs			860	1377
Total loci with evidence of expression			2032	2766

We analysed whether our data contained transcribed regions of the genes involved in stress response, pathogen resistance, and flowering and the genes encoding seed storage proteins and some enzymes reported to be located on wheat chromosome 6B. The α-gliadin gene (acc. no. JX141494), the stripe rust resistance gene *Yr36* (EU835199), and the grain protein content gene *Gpc-B1* (DQ869673) were mapped to contigs from 6BS, and the α-amylase gene (M16991) and the genes for three low-temperature-responsive dehydrins, *Wcs120* (M93342), *Wcs66* (L27516), and *Wcor410* (L29152), were mapped to 6BL contigs. These results for the chromosomal assignment of known genes were in accordance with previous studies.^25,50,51^ The three homoeologous sequences containing the flowering time genes, *TaHd1-1*, *TaHd1-2*, and *TaHd1-3* had been isolated from the long arm of chromosomes 6A, 6B, and 6D, respectively.^52^ Our analysis reconfirmed that the sequence of *TaHd1-2* (AB094488) was mapped to a contig from 6BL with 100% identity, which is in good agreement with the previous report. We also examined whether the genes isolated from chromosomes 6A or 6D but not from 6B were homoeologous on chromosome 6B. The gene involved in vernalization, *TmVIL2* (vernalization insensitive 3-like 2) (DQ886917), has been isolated from the diploid wheat *Triticum monococcum* and was mapped to the short arm of chromosome 6A^m^.^53^ We found a contig containing the whole gene sequence of *VIL2* on 6BS (Supplementary Fig. S2), which indicates that the homoeologous gene copy of *TmVIL2* is conserved on chromosome 6B in hexaploid wheat. These mapping data support our survey sequences of wheat chromosome 6B, which contain previously reported genic regions and are useful for mining the genes located on chromosome 6B.

### 3.5. Identification of the genes for functional non-protein-coding RNAs

The short arm of wheat chromosome 6B is characterized by the presence of satellite and a secondary constriction, NOR (nucleolus organizer region), as is chromosome 1B,^22^ which features a rDNA locus that contains approximately 5500 rRNA genes.^26^ To explore the structure of the rDNA region, we searched for contigs with homology to sequences corresponding to the 5S, 5.8S, 18S, and 25S rDNAs and a spacer sequence between 18S and 25S. From the 6BS assembled sequences, we found only eight contigs that showed homology to any sequence with >95% identity and >100 bp alignment. However, seven of these contigs exhibited extremely high read depths (73.7–203.8) (Table 3). Because the average read depth observed for 6BS was 9.05, the high depth rates indicate that these contigs represent sequences with high copy numbers. This result demonstrates that rDNA regions, including spacer sequences, were assembled in a few contigs because of high sequence similarity under functional constraint. In general, spacer sequences are more diverse than rDNA sequences because of a low functional constraint. In our study, the spacer sequences contained four repeat families that can be a source of additional diversification.^54^ Nevertheless, the rDNA regions consistently have high read depths in our survey sequencing, which suggests that concerted evolution occurred in this region, although the possibility that the rDNA regions were duplicated recently cannot be excluded.

**Table 3.** Contigs containing rDNA on the short arm of chromosome 6B

Contig	Query	Identity (%)	Contig length (bp)	Query length (bp)	Alignment length (bp)	Read depth
Contig254561	25S	98	231	3450	228	202.7
Contig172978	18S	99	505	1869	303	188.7
Contig112653	5.8S	96	722	223	164	121.1
Contig113562	Spacer	100	718	4642	718	203.8
Contig254561	Spacer	98	231	4642	231	202.7
Contig177561	Spacer	99	489	4642	489	140.2
Contig225039	Spacer	100	322	4642	322	73.7
Contig53095	Spacer	98	1136	4642	248	2.7

**Figure 5.**
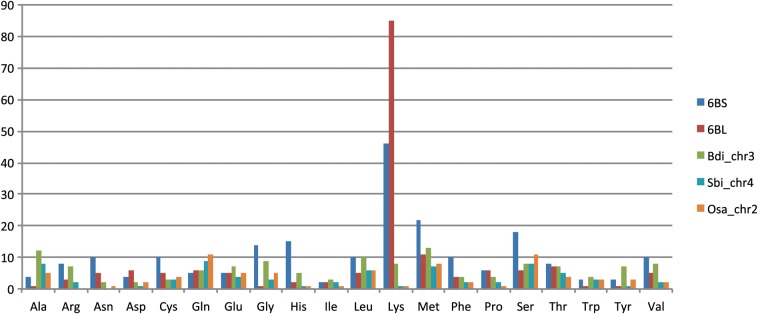
The prediction of tRNA species on chromosome 6B. The number of tRNAs detected by tRNAscan-SE ver. 1.3.1 was counted. Pseudogenes were excluded, and tRNAs covered by repetitive elements were included.

We detected 213 and 167 predicted tRNA genes in 6BS and 6BL, respectively. In both chromosome arms, the tRNA^Lys^ gene was the most abundant, followed by the genes for tRNA^Met^ (Fig. 5). Such a skewed distribution of tRNA genes was not observed in any of the syntenic chromosomes to wheat chromosome 6 in other grass species, e.g. chromosome 2 of *Oryza sativa*, chromosome 3 of *B. distachyon*, and chromosome 4 of *Sorghum bicolor*. We hypothesized that the expansion of a particular tRNA gene could be caused by repetitive elements, that is, if one tRNA gene is located in a repetitive region, the copy number of the gene increases dramatically along the propagation of the repetitive sequence. As expected, 83 of 131 tRNA^Lys^ genes on chromosome 6B were located in an LTR retrotransposon, Gypsy, or *de novo* repeats. Although the details of the *de novo* repeats containing tRNA^Lys^ are not clear, tRNA expansion could occur through repetitive elements. Because 86.7% of tRNAs located in repeat regions were tRNA^Lys^ or tRNA^Met^, the skewed distribution of the tRNA genes on wheat chromosome 6B can be explained by the expansion of repetitive elements containing specific tRNA genes.

miRNAs are a class of small RNAs that mediate gene silencing at the post-transcriptional level.^55^ Only 42 wheat miRNAs are stored in miRBase as of release 19,^37^ which is significantly fewer than the number for other grass plants (rice: 708, maize: 321, sorghum: 242, and *Brachypodium*: 136). These observations suggest that more miRNA genes remain to be identified in the wheat genome. To find known/novel miRNA genes in our assembly, we conducted a homology search in miRBase using known plant miRNAs. A total of 2906 miRNAs (1381 loci on 6BS and 1525 loci on 6BL) were predicted using 350 mature plant miRNAs in miRBase as queries (Table 4). Some miRNAs are located in repeat regions,^14,16^ as has been observed for tRNA genes. Consistent with these previous reports, all but 26 of the predicted miRNA genes are located in repeat-masked regions. Especially, 1805 miRNA genes were located in a DNA transposon, Mariner, and 766 genes were located in CACTA repeats. Even though the LTR retrotransposons Gypsy and Copia were distributed most widely on both arms, only 63 miRNA genes were located in these transposons. These results indicate that miRNA genes propagate in the wheat genome with the diffusion of specific transposons, although which of the predicted miRNA genes are transcribed into mature miRNAs has not been determined.

**Table 4.** Putative miRNA species identified in the survey sequences of the short and long arms of chromosome 6B

	6BS	6BL	Both
Locus	1381	1525	
Wheat miRNA evidence	825	913	
Non-wheat miRNA evidence	556	612	
Number of hit query	205	204	350
Identical locus to query miRNA	146	175	
Number of miRNA for identical locus (wheat)	10	12	13
Number of miRNA for identical locus (non-wheat)	4	8	8

### 3.6. Comparative analysis of syntenic chromosomes to wheat 6B among monocot species

The barley genome sequence was recently reported,^46^ and the annotation data for this species can be used as a possible gene set as the closest relative of wheat. Of the 26 159 high-confidence genes in barley, 2573 loci are located on chromosome 6H. Mapping barley genes to our wheat 6B assembly revealed that 2399 genes had significant hits on 2070 loci of wheat 6B (*E*-value < 10^−5^). Chromosome arm information is available for 423 and 313 genes located in chromosome 6HS and 6HL, respectively; therefore, we compared these barley genes with the arm information of our wheat assembles. We found that 380 of 423 6HS genes (89.8%) mapped to chromosome 6BS, and 246 of 313 6HL genes (78.6%) mapped to chromosome 6BL. Based on these data, we concluded that our assemblies provide good coverage of the transcribed regions, which were supported by the synteny with barley chromosome 6H. However, chromosome arm information is still missing for more than two-thirds of the genes on 6H; therefore, we cannot analyse the syntenic relationship between wheat 6B and barley 6H more precisely.

**Figure 6.**
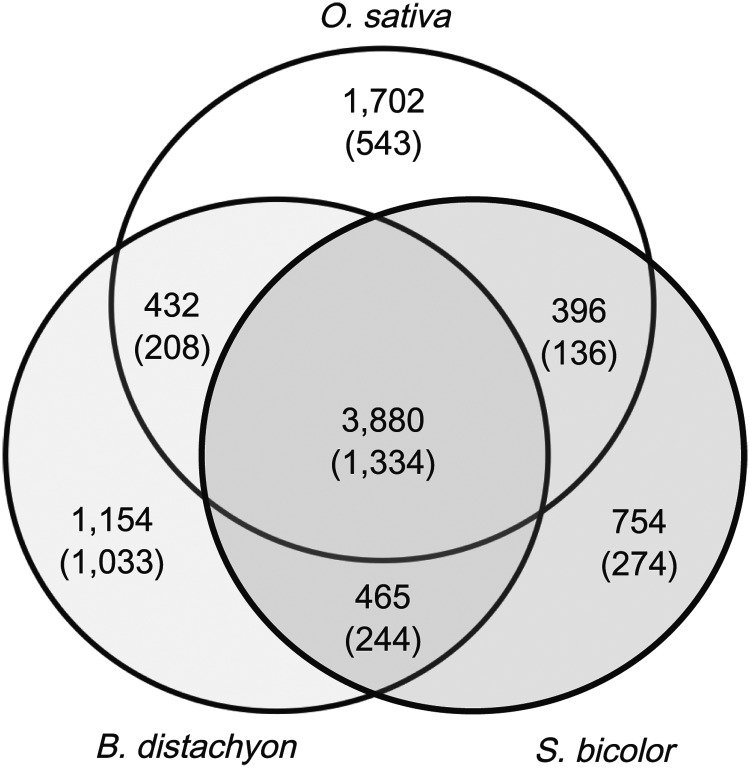
The distributions of the genes found on chromosome 6B with significant similarity to *O. sativa*, *B. distachyon*, and *S. bicolor*. The numbers in parentheses represent loci on which genes from syntenic chromosomes were mapped.

We also compared the wheat 6B assemblies with the annotation data for monocot plant species such as *O. sativa*, *B. distachyon*, and *S. bicolor* to identify homologous regions. Our search indicated that 8783 loci from the three monocot plants were possibly homologous to wheat 6B contigs, and 3880 of which were found in all four genomes (Fig. 6). Wheat homoeologous group 6 chromosomes have a synteny with chromosome 2 of *O. sativa* (Os02), chromosome 3 of *B. distachyon* (Bradi3), and chromosome 4 of *S. bicolor* (Sb04).^56,57^ Our results demonstrated that 3772 loci were syntenic to at least one of the syntenic chromosomes of the three monocot plants. The mapping ratio of syntenic genes (40.2–59.7% between our annotated loci and those of syntenic chromosomes in the other three species) was comparable with the total coverage of our assembly for wheat chromosome 6 (55.6%).

To verify the reliability of these loci, we assessed the wheat transcriptome evidence, such as wheat FLcDNA/mRNAs and predicted genes with EST evidence. We found that 57.4% of the syntenic regions had transcriptome evidence, which was significantly higher than the value for non-syntenic regions (32.7%). In particular, the regions syntenic to all three monocot species were highly supported by the transcriptome data (79.9%). These results confirmed that wheat chromosome 6 has conserved synteny with the chromosomes of other grass species at the sequence level.

## 4. Conclusions

Here, we have provided the whole-chromosome shotgun sequence of wheat chromosome 6B, which provides an overview of the sequence features of this chromosome, including rDNA regions, a characteristic structure of wheat 6B, and we present new information about the TEs, expressed genes that are syntenic in other phylogenetically related species, and non-protein-coding tRNA and miRNA genes. We are now conducting on the reference genome sequencing of chromosome 6B using the MTP BACs within the framework of the IWGSC. However, filling the sequence gaps and evaluating the quality of the assembly data using only one data set may be difficult. This survey sequence provides valuable information for completing the genome assembly as well as the mate-pair sequencing, which is also now underway. Furthermore, the survey sequence information in this study will be directly used to identify 6B genes that can be exploited to control agronomically important traits and to construct DNA markers for these traits. The assembled contigs will be available for browsing on our web site (KomugiGSP; http://komugigsp.dna.affrc.go.jp/index.html, 20 September 2013, date last accessed).

## Supplementary Data

Supplementary Data are available at www.dnaresearch.oxfordjournals.org.

## Funding

This work was supported by the Ministry of Agriculture, Forestry, and Fisheries of Japan (Genomics for Agricultural Innovation, grant number KGS-1003 and -1004), the Czech Science Foundation (award P501/12/G090), the Ministry of Education, Youth and Sports of the Czech Republic, and the European Regional Development Fund (Operational Programme of Research and Development for Innovations No. ED0007/01/01).

## Supplementary Material

Supplementary Data
