# Supplementary material for: Next-Generation Survey Sequencing and the Molecular Organization of Wheat Chromosome 6B
Source: DNA Res. 2013 Oct 1;21(2):103–14. doi: 10.1093/dnares/dst041 (PMC3989483; doi:10.1093/dnares/dst041)
Supplement: Supplementary Data [file supp_21_2_103__index.html]

Next-Generation Survey Sequencing and the Molecular Organization of Wheat Chromosome 6B — Supplementary Data 

# Next-Generation Survey Sequencing and the Molecular Organization of Wheat Chromosome 6B

## Supplementary Data

Supplementary Data

**Files in this Data Supplement:**

- Supplementary Data - Pdf file
