# Supplementary material for: Next-Generation Survey Sequencing and the Molecular Organization of Wheat Chromosome 6B
Source: DNA Res. 2013 Oct 1;21(2):103–14. doi: 10.1093/dnares/dst041 (PMC3989483; doi:10.1093/dnares/dst041)
Supplement: Supplementary Data [file supp_dst041_dst041supp.pdf]

Supplemental Table 1. The summary of the FISH analysis of the transposon sequences on chromsome 6B.

					Forward primer		Reverse primer				FISH	
Type	Class	Family	Primer number	Sequence used for primer designen (GenBank Acc. #)	Position	Sequence	Position	Sequence	Expected amplicon size	Amplification	Uniquitous labeling	Localization on 6B
Type I	Gypsy	nusif	1	FN564426	220823-220842	GAGGAGGCGGAGAGATCAAG	220929-220948	GCACCAAAGAGGAAACAAGG	128	Discrete	Yes	Dispersed
		egug	1	HE774675	154914-154933	AGTAGTTGCGGATGCTTGCT	154787-154806	GAGTTGCGGAATTGTCCCTA	156	Discrete	Yes	Dispersed
		derami	1	FN564426	1500515-1500537	GGTTTTTGTTGAGTTTTGTGTGA	1500388-1500407	CATCCCCAAGCTTAGTTGCT	154	Discrete	Yes	Dispersed
		laura	1	FN564430	1792915-1792934	TGCGGTAGTTGAATCCCTTT	1792744-1792763	GTTCACACACCTCCGATTCA	194	Discrete	No (2 genomes)	Dispersed
		fatima	1	FN564428	2507167-2507186	AAACCGGATGGGTTTAGTCC	2507342-2507361	GAGACAGGGGACACGATGTT	205	Discrete	No (2 genomes)	Dispersed
			2	FN564428	2507167-2507186	AAACCGGATGGGTTTAGTCC	2507329-2507348	CGATGTTTTACCCAGGTTCG	192	Discrete	No (1 genome)	Dispersed
		sabrina	1	JF701619	146285-146304	TTGATGTATCGCGAGCTTTG	146011-146030	TGCCCCAAAGTTTCTACCAG	299	Discrete	No (2 genomes)	Dispersed
			2	FJ447464	56575-56594	ACCATCTCTTCGCCGAACTA	56723-56742	TTTCCCTCAAGTGGATGACC	167	Discrete	No (2 genomes)	Dispersed
			3	HE774676	24311-24330	TCGAGATCCGATGAATTGTG	24505-24524	CCCCTACTGTCACTGGGAAA	213	Discrete	No (2 genomes)	Dispersed
			4	FN564431	57459-57478	ATCCCCTCTTTCGTCTTCGT	57574-57595	CAGCAAGGATGATTGTGGTAAA	136	Discrete	No (2 genomes)	Dispersed
		wham	1	FN564430	998301-998320	TCGCGTTTTACGATACTGGA	998142-998161	CAGCAAGCCTACGATGGAAT	179	Discrete	Yes	Dispersed
	Copia	angela	1	HE774676	1156500-1156519	ACATGACCCATTCCGTTAGC	1156605-1156624	AAGGTGTTCCTCCGGTATCC	126	Discrete	Yes	Dispersed
			2	FN564427	729827-729846	CCAGGTTCCGCTATTGGTTA	729611-729630	TTCCGGTGTCCGAATATAGC	241	Discrete	Yes	Dispersed
Type II	CACTA	clifford	1	FN645450	1258990-1259009	CCGGAGGAGAAGGTAGATCC	1258915-1258934	ATGCGCTCATAGTTGCCTTT	99	Discrete	Yes	Dispersed
		jorge	1	FN564436	572271-572290	GACCGTAGTGCTCCAACACA	572187-572206	GGAGGAAACGGACCTCCTAC	104	Smear	Yes	Dispersed
		pavel	1	FN564430	1109949-1109968	AAACCACCTCTGTGGTCAGG	1109710-1109729	GCAAGCTTCACATGCAACAT	257	Discrete	Yes	Dispersed
		wilma	1	FN564434	1269040-1269059	GGTGCTTGTGGGTTGCTAGT	12496-12515	GACTTCTCCCCATGTCCTCA	174	Discrete	Yes	Dispersed
		caspar	1	AM932684	29857-29876	CGTACCATGCATGACACCTC	29757-29776	CTTGGGCGATCGTGTAAAAT	123	Discrete	No (2 genomes)	Dispersed
			2	FN564434	1168185-1168204	CAGTGCGGAGTTCTTGTGAA	1167999-1168018	CGCCATCTTCCTAAAAGCAG	206	Discrete	No (2 genomes)	Dispersed
	Mariner	athos	1	HQ391329	1- 24	ACAACATACTACTCCCTCTGTCCC	242-261	GCAGTTGGGCACAGTTGGCT	260	Non discrete	Yes	Dispersed
		icarus	1	HQ703016	1258-1277	AGAACTGGTGTAGCCGAGGA	1448-1467	GATTGCAGCCACAACTCTGA	209	Smaller	No (2 genomes)	Dispersed
		thalos	1	AK335477	879-897	CTGTTAGCAGCGGGACAAG	1090-1113	GCAATAAAGAGTGAAATAGAAGC	234	Smaller	Yes	Dispersed
	Harbinger	kerberos	1	FN564430	845136-845155	AGAGAAGGTAAGCGGGCTCT	845283-845302	TAAGCACTTGCCATGTCACC	171	Discrete	No (2 genomes)	Dispersed
unknowr	unknown	xc	1	AK331835	469-487	GCCTTAGCACGACGACTTC	565-584	GCGACGACTACAAGCACTGA	115	Discrete	Yes	Dispersed

Transposons present in the 6B survey sequences were arbitoralily selected. They belong to Type I (retrotoransposon) or Type II (DNA transposon) . Type I transposons were further classified to Class and Family according to the sequence homology to the TREP, the Triticeae Repeat Sequence Database (http://wheat.pw.usda.gov/ITMI/Repeats/). For each transposon family, Genbank accession number of the sequence used for primer design, position of primer, primer sequence, and results of PCR and FISH are given. The result of PCR indicate whether PCR primers gave the discrete band. The FISH results indicate the hybridization pattern in terms of hexaploid genomewide ("Yes" indicate dispersed on three genomes, "No" indicate the genome specific hybridization with number of the positive genomes in parentheses) and distribution on the 6B chromosome.

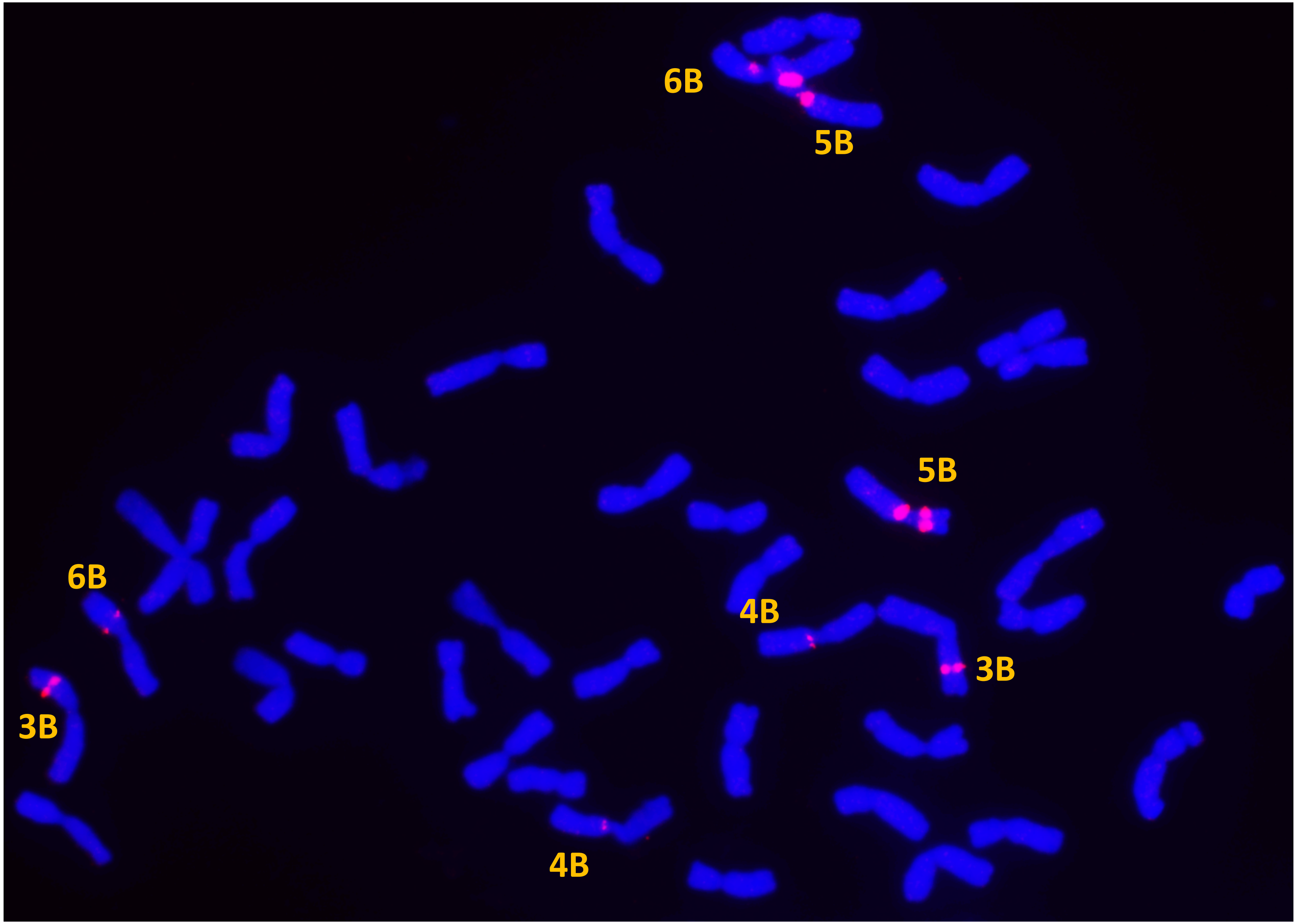

Supplemental Figure 1. The FISH pattern of the mitotic metaphase chromosome probed with the AG_{12} satellite sequence. The probed region (shown in red) was detected by Cy3-Streptavidin (GE Healthcare Life Sciences, USA), and the chromosomes were counterstained with DAPI (4',6-diamidino-2-phenylindole, shown in blue). Note that chromosome 6B can be identified by the presence of satellite and AG_{12} signals on the short arm.

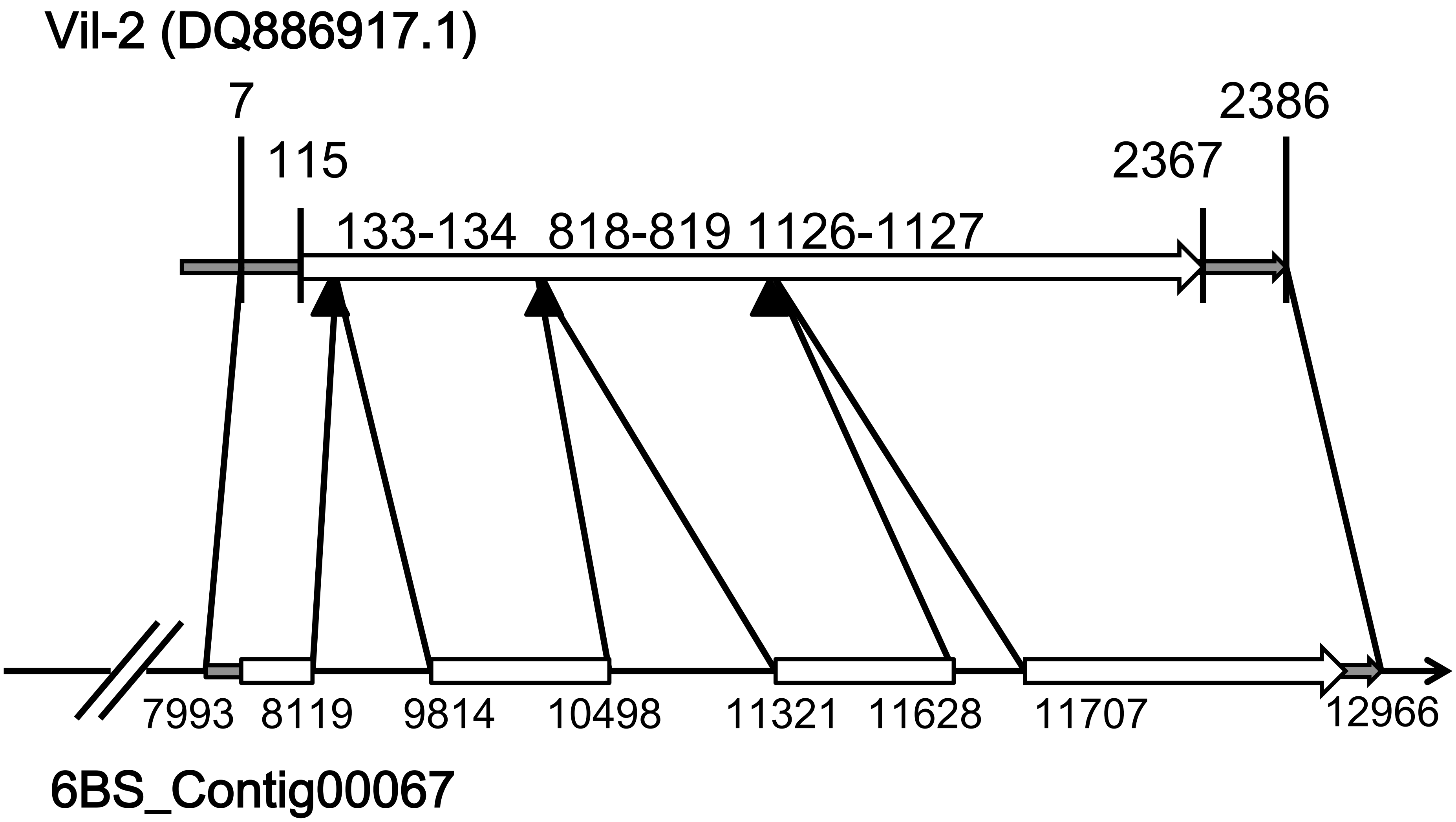

Supplemental Figure 2. The gene structures of *TmVIL2* and its orthologue on the short arm of chromosome 6B. The positions of the alignable regions and introns are given. The grey arrows represent the UTRs, and the white boxes represent the CDSs.
